# Couples experiences of receiving uncertain results following prenatal microarray or exome sequencing: A mixed‐methods systematic review

**DOI:** 10.1002/pd.5729

**Published:** 2020-05-24

**Authors:** Eleanor Harding, Jennifer Hammond, Lyn S. Chitty, Melissa Hill, Celine Lewis

**Affiliations:** ^1^ BSc Paediatrics and Child Health The UCL Great Ormond Street Institute of Child Health London UK; ^2^ London North Genomic Laboratory Hub Great Ormond Street Hospital for Children NHS Foundation Trust London UK; ^3^ Genetics and Genomic Medicine The UCL Great Ormond Street Institute of Child Health London UK

## Abstract

**Background:**

Tests in pregnancy such as chromosomal microarray analysis and exome sequencing are increasing diagnostic yield for fetal structural anomalies, but have greater potential to result in uncertain findings. This systematic review investigated the experiences of prospective parents about receiving uncertain results from these tests.

**Methods:**

A systematic search of three electronic databases was conducted. Data extraction was performed for studies that met the eligibility and quality criteria. Results were synthesised following the principles of thematic analysis.

**Results:**

Fourteen studies (10 qualitative, 4 quantitative) were included. Findings were grouped into three overarching themes. *Sources of uncertainty* included the testing procedure, the diagnosis and prognosis, and health professionals' own uncertainty. The *clinical impact of the uncertainty* included parents struggling to make clinical decisions with the information available, the *emotional impact* included decisional‐regret, shock, worry and feeling overwhelmed. To manage the uncertainty, parents sought support from healthcare professionals, friends, family, the internet and other parents as well as remaining hopeful.

**Conclusions:**

Prospective parents experience a myriad of uncertainties in the prenatal setting, which must be handled sensitively. Future research should explore optimal ways of managing uncertainty to minimise harm. Recommendations are made for discussing uncertainty during pre‐ and post‐test counseling.

**What is already known about this topic?**

- Couples often choose chromosomal microarray and exome sequencing during pregnancy in anticipation of reassurance about the health of the fetus, but sometimes receive uncertain results.

**What does this study add?**

- Here we synthesise the current research on parents' experiences of receiving uncertain results in pregnancy including the sources of uncertainty, clinical and emotional impact of uncertainty and how uncertainty is managed.

## 1 BACKGROUND

Fetal anomalies occur in 2% to 5% of pregnancies and cause around 21% of perinatal deaths.^1^, ^2^, ^3^ Initially, prenatal testing for fetal anomalies was limited to karyotyping and targeted genetic testing.^4^ Chromosomal microarray analysis (CMA), which is able to evaluate the sub‐microscopic structure of chromosomes is now being offered routinely in many countries, and prenatal exome sequencing (ES), which provides resolution down to the single base‐pair, is beginning to be used clinically to increase diagnostic rates.^5^ There are a number of benefits in getting a result from prenatal testing. This includes the potential to provide a definitive diagnosis during pregnancy which can then inform genetic counselling, pregnancy and delivery management, and pre‐ and post‐natal care.^6^, ^7^

Whilst genomic technologies such as CMA and ES increase the number of genetic diagnoses made in pregnancy, there remain practical and ethical challenges in interpreting results in a way that is meaningful for parents.^8^ Furthermore, tests such as CMA and ES have a greater potential to result in uncertainty.^9^, ^10^, ^11^ This is particularly challenging in the prenatal setting as many parents enter into prenatal testing hoping for and expecting reassurance and may use prognostic information to make a decision about pregnancy termination.^12^ Uncertainty may arise for a number of reasons. There may be uncertainty due to a variant of uncertain significance (VUS) being identified where the relevance of that variant to the health of the baby is unknown. ^10^ Some conditions have variable expressivity, incomplete penetrance or fetal phenotype information may be limited meaning that even where a variant is known to be significant, it is not possible to predict the prognosis.^13^, ^14^ If no significant variant is found following an abnormal ultrasound, parents may feel they are still in a state of uncertainty around the health of the baby.^15^

The last decade has seen a number of studies looking at prospective parents' experience of uncertainty in the prenatal setting. Parents frequently state that they are interested in receiving uncertain results but are surprised when they receive them,^16^, ^17^ sometimes experiencing shock, confusion and anxiety.^18^, ^19^ Here, we describe a systematic review to synthesise the literature around parents' experience of receiving uncertain results in pregnancy following CMA or ES.

## 2 METHODS

### 2.1 Ethical approval

Ethical approval was not required for this study.

### 2.2 Design

We have undertaken a systematic review to bring a formal structure to the identification, evaluation and synthesis of research findings. As qualitative, quantitative and mixed‐methods studies have been sought, an integrative approach to data synthesis has been used.^20^

### 2.3 Search strategy

**FIGURE 1:**
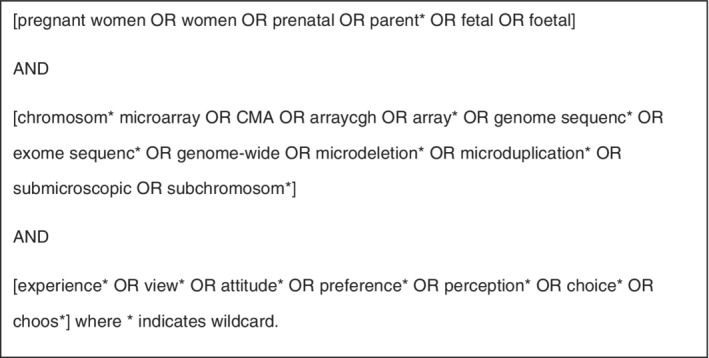
Search terms used to identify studies

A systematic search was conducted across three electronic databases (PubMed, Embase, PsycINFO), using the search terms in Figure 1. The reference lists of eligible studies were searched, as well as other studies by the first named author. The initial search was conducted in October 2018. A further search was conducted in July 2019 and no additional papers were identified.

### 2.4 Study selection

**FIGURE 2:**
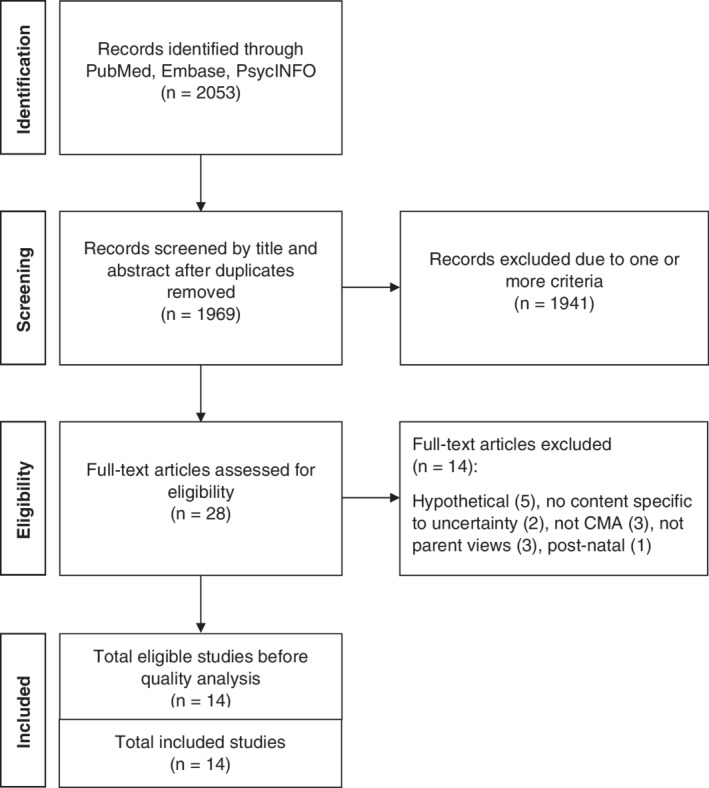
Study selection process (PRISMA flow diagram)

The study selection process followed the Preferred Reporting Items for Systematic Reviews and Meta‐Analyses (PRISMA) guidelines (Figure 2).^21^ Following the removal of duplicates, titles and abstracts were independently reviewed against the inclusion/exclusion criteria by two researchers. The full text of any potentially relevant studies were retrieved for further review and considered against the inclusion and exclusion criteria independently by three researchers. Any discrepancies regarding study inclusion were discussed until consensus was reached.

Studies were included if they were:

1. Investigating pregnant women and partners' experiences of uncertainty through the process of having CMA or ES;
2. Using qualitative, quantitative, cross‐sectional or mixed‐methods research approaches;
3. Published in English in a peer‐reviewed journal.

Studies were excluded if they were:

1. Investigating experiences of uncertainty not identified following CMA or ES, such as risk scores following Down syndrome screening, non‐invasive prenatal testing or karyotyping;
2. Investigating parents' experiences following newborn or paediatric CMA and ES;
3. Examining views of uncertainty based on purely hypothetical scenarios;
4. A review, case report, abstract, editorial or commentary.

### 2.5 Quality assessment

The eligible studies were critically appraised for biases using the standard quality assessment criteria developed by Kmet et al, which allows the assessment of both qualitative and quantitative research.^22^

Eligible studies were appraised by two researchers (E.H. and M.H.). Checklists for qualitative (10 criterion) and quantitative (14 criterion) studies are scored as ‘met’ (2 points), ‘partially met’ (1 point), ‘not met’ (0 points) or ‘not applicable’. The total score is converted to a percentage. We used a low cut‐off point of 55%, described as liberal by Kmet et al and following the approach of other mixed methods systematic reviews.^22^, ^23^

### 2.6 Data extraction and synthesis

Study details, including the aim, study design, demographics and findings, were extracted into a summary table (Table 1). NVivo12 software was used to facilitate coding and analysis.^24^ The quantitative and qualitative data were analysed using the principles of thematic analysis.^20^, ^25^ The results section of each of the studies which related to the experience of receiving an uncertain result was coded. For qualitative studies direct quotes from participants, themes and descriptions were coded. For quantitative studies tabulated data and descriptions of findings were coded. For our thematic analysis, a codebook was initially developed by three researchers (E.H., M.H. and C.L.) who independently coded two randomly selected included studies. The codes were compared and discussed until a consensus was reached. At this stage, codes that were similar were grouped into broad categories, which were then refined and grouped into overarching themes.

**TABLE 1:** Summary of included studies

Reference/country	KMET score (%)	Aim	Method	Participants	Findings and key themes relating to uncertainty before and after receiving CMA and ES results
Bernhardt et al, USA ^15^	80	Explore the experiences of women receiving abnormal results from prenatal CMA testing performed in a research setting	Qualitative Semi‐structured telephone interviews	23 women who had received positive/uncertain CMA results	After receiving uncertain results from CMA, participants were shocked, anxious and overwhelmed as they struggled with the lack of a definitive answer. The majority of participants felt they were not properly informed of the possibility of uninterpretable or uncertain results.
Desai et al, USA ^29^	86	Examine the influence that the return of genomic (CMA) results had on parental well‐being and perceptions of children's development	Quantitative 12 study instruments	138 participants (80 mothers, who underwent prenatal CMA, and 58 fathers) Mean age: 38.1 years	Participants who received VUS rated their child as less competent at 12 months than parents who received normal/likely benign test results (*P* = .04) but at 36 months there was no difference (*P* = .37), using the Brief Infant‐Toddler Social and Emotional Assessment instrument. At 36 months, participants who received VUS were significantly less satisfied with their decision to undergo genetic testing compared to the group who received normal results (*P* = .02).
Halliday et al, Australia ^34^	91	Examine the choices pregnant women make about the amount of foetal genetic information they want from CMA	Quantitative 2 self‐administered surveys with validated scales	111 pregnant women who underwent invasive prenatal testing, without a foetal structural abnormality	A slight majority (59.5%) of participants chose to receive the extended analysis report, which included VUS, over the targeted analysis report, which included only abnormalities with 100% penetrance.
Hillman et al, UK ^30^	85	Gain insight into the experiences of women and their partners diagnosed with a foetal abnormality on prenatal ultrasound examination and receiving CMA testing	Qualitative Semi‐structured interviews	25 women after receiving CMA results (12 male partners were present) Median age: 29.6 years	Participants felt uncertain during the long wait for results as they were aware of the 24‐week pregnancy termination time‐limit. Participants struggled with uncertainty within the medical team, when a healthcare professional was unable to give them the answers they wanted.
Quinlan‐Jones et al, UK ^19^	85	Explore parental experiences of ES for prenatal diagnosis and explore their reasons for undergoing genomic testing	Qualitative Semi‐structured interviews	12 women who underwent ES testing for prenatal diagnosis Mean age: 28.7 years	Participants reported a ‘fear of the unknown’ due to uncertainty for the baby. Participants were uncertain about the process their results would be returned and wanted this better explained to them.
Robson et al, UK ^31^	80	Determine what factors influence parents' and health professionals' choices and decision‐making about CMA	Qualitative sub‐study Semi‐structured interviews	16 women following CMA testing (5 male partners were present) Mean age: 35.3 years	The majority of participants did not recognise that CMA results could introduce more uncertainty. One participant reported that individuals have differing tolerances for uncertainty.
Rubel et al, USA ^27^	85	Assess how participants receiving abnormal prenatal genetic testing results seek information and understand the implications of results	Qualitative Semi‐structured telephone interviews	39 participants following abnormal/VUS results (27 women age range: 20‐43 years, 12 male partners age range: 29‐46 years)	Many participants found themselves in a ‘state of shock’ on receiving an uncertain CMA result. Many participants struggled to quantify and interpret the risks associated with their uncertain results. Some wished they did not have the information at all.
van der Steen et al, The Netherlands ^32^	85	Examine pregnant couples' preferences, doubts and satisfaction regarding the scope of invasive prenatal diagnosis	Quantitative Pre‐test self‐reported survey, post‐test interviews (validated measures)	250 participants (141 pregnant women who underwent prenatal CMA testing and 109 male partners) Mean age: 38.8 years	84% and 44% of participants in the prenatal screening and prenatal diagnosis groups respectively wished to be informed of significance loci if detected, despite the uncertainty they might have brought. Participants who opted for results of a lower resolution experienced significantly more doubt while waiting for results.
van der Steen et al, The Netherlands ^33^	80	Evaluate the psychological impact of disclosing a prenatal diagnosis of susceptibility loci	Qualitative Semi‐structured interviews	12 participants (8 women who underwent prenatal CMA testing and 4 male partners) Age range: 23‐41 years	Participants reported feeling shocked when they were told about a susceptibility loci, and subsequently feeling uncertain about the prognosis for the child and future. Participants expressed a desire to receive as much information as possible, despite the possibility of receiving unclear results with an uncertain prognosis.
Walser et al, USA ^17^	90	Identify women's specific pre‐ testing and post‐testing informational needs, as well as their preference for return of various types of results	Quantitative Survey	155 women who underwent prenatal CMA testing	Participants expressed the importance of pre‐test counselling to aid understanding of the test. Participants thought it was important that all types of results were reported, including VUS.
Walser et al, USA ^26^	90	Explore how couples' understanding of CMA results impacts their decision‐making and level of concern about the clinical implications of the results	Qualitative Semi‐structured interviews	40 participants (28 women, who had received abnormal/uncertain CMA results and 12 male partners) Mean age: 35.5 years	Participants were relieved to discover an inherited variant following a VUS finding, as they found comfort in knowing that the carrier parent was ‘reasonably healthy’. Most participants who received VUS struggled with the lack of information, especially regarding the spectrum of severity for the variant.
Werner‐Lin et al, USA ^18^	85	Examine couples' understanding and incorporation of findings into decision making regarding pregnancy termination following CMA	Qualitative Semi‐structured telephone interviews	24 participants (12 women who had received a pathogenic/VUS result and 12 male partners) Mean age: 35.5 years	Participants described feeling numb and dazed when they received VUS results, as well as being unaware of the possibility of uncertain/inconclusive results. Many participants wanted support from healthcare professionals. Some participants wanted support from others in a similar situation.
Werner‐Lin et al, USA ^28^	85	Understand how, in the wake of prenatal CMA testing which identified a copy‐number variant of uncertain or variable significance, mothers interpret, and make meaning of the variant through early infancy	Qualitative Semi‐structured interviews	23 women who had received prenatal CMA results Mean age: 36 years	Participants who received VUS sought services to assess their child's development over time. The uncertainty left them worrying about how to best support their child. Participants who had children with serious health issues reported not thinking about the uncertain result at all with everything else that was going on.
Wou et al, USA ^6^	90	Investigate the experiences of couples who underwent prenatal ES for fetal anomalies and the amount/type of information couples want from prenatal ES	Qualitative Semi‐structured interviews	29 participants (17 pregnant women who underwent ES and 12 male partners) Mean maternal age: 33.9 years	Participants reported feeling anxious, stressed and overwhelmed whilst waiting for results, due to the long wait for ES results. Participants wanted support from a known healthcare provider, as well as other families going through the same thing.

Abbreviations: CMA, Chromosomal microarray analysis; ES, exome sequencing.

Once all the studies were coded, the researchers reviewed each of the codes, categories and themes and some minor changes were made (eg, splitting or combining codes, renaming themes).

## 3 RESULTS

Titles and abstracts for 1969 studies were identified, following removal of duplicates, and independently reviewed against the inclusion/exclusion criteria by two researchers. The full text of 28 studies were retrieved for further review by E.H., M.H. and C.L. independently and any discrepancies regarding study inclusion were discussed. Of these 28 studies, 14 were excluded. Quality appraisal scores of the included studies ranged from 80% to 91% (Table 1). All 14 eligible studies exceeded the 55% cut‐off point and were included in the review.

### 3.1 Study characteristics

Fourteen studies representing the views of 914 participants (678 women, 236 partners) were included in the review (Table 1). Eight studies were from the USA,^6^, ^15^, ^17^, ^18^, ^26^, ^27^, ^28^, ^29^ three were from the UK,^19^, ^30^, ^31^ two were from the Netherlands,^32^, ^33^ and one was from Australia.^34^ Twelve studies investigated the experiences of women and partners who underwent CMA,^15^, ^17^, ^18^, ^26^, ^27^, ^28^, ^29^, ^30^, ^31^, ^32^, ^33^, ^34^ and two studies investigated the experiences of those who underwent ES.^6^, ^35^ Six studies exclusively explored experiences after the test results were returned,^18^, ^26^, ^27^, ^28^, ^29^, ^33^ while the remaining eight studies also investigated experiences whilst waiting for the results.^6^, ^15^, ^17^, ^19^, ^30^, ^31^, ^32^, ^34^ Methodological approaches included 10 qualitative studies,^6^, ^15^, ^18^, ^19^, ^26^, ^27^, ^28^, ^30^, ^31^, ^33^ and four quantitative studies.^17^, ^29^, ^32^, ^34^ The types of uncertain results participants received included uncertainty related to VUS,^17^, ^26^, ^27^, ^29^, ^30^, ^31^, ^34^ deletion/duplication syndromes,^15^, ^18^, ^26^ susceptibility loci,^32^, ^33^ copy number variants,^28^ and negative ES results.^6^

The criteria for offering CMA/ES and parents reasons for having these tests differed across the 14 studies, including: an abnormal ultrasound in the first or second trimester,^6^, ^15^, ^17^, ^18^, ^19^, ^26^, ^28^, ^29^, ^30^, ^31^ advanced maternal age,^15^, ^17^, ^18^, ^26^, ^28^, ^29^, ^32^, ^34^ family history of genetic abnormality,^17^, ^18^, ^26^, ^28^, ^29^, ^34^ positive serum screen,^15^, ^17^, ^18^, ^26^, ^28^, ^29^, ^34^ maternal request,^34^ a previous child with a genetic or chromosomal abnormality,^17^, ^18^, ^26^, ^28^ parent(s) a carrier of a chromosome deletion or duplication,^17^ all indications of increased risk of aneuploidy in cases without ultrasound abnormalities^33^ and a desire for more information.^18^ The reasons in one paper were not stated.^27^

Three overarching themes relating to uncertainty following CMA/ES results emerged during analysis and are described below.

#### 3.1.1 Sources of uncertainty

Sources of uncertainty included women and partners' uncertainty around the testing procedure itself, uncertainty about what the results meant including the diagnosis and prognosis, uncertainty about whether online information was accurate, and healthcare professionals' (HCPs) uncertainty. More detail is provided in Table 2.

**TABLE 2:** Themes relating to sources of uncertainty

Themes	Example quote/findings
**Testing procedure**
Not knowing the test could reveal uncertain results ^18^ Uncertainty about the test itself^15^, ^18^, ^31^ Uncertainty around who delivers the results and how they are delivered^6^, ^19^	‘I was not aware that we could get inconclusive results, or they would find something, but it not mean anything to them’. *[Patient quote]* ‐^18^ Because it seemed risk‐free, many women said they had not understood much about microarray testing before having it done *[Findings]*. ‐^15^ Some parents were uncertain regarding the process by which results would be returned and would have appreciated having this better explained to them. Some parents preferred to return to the hospital and have the results explained by familiar clinicians face to face. *[Findings]* ‐^19^
**Results, including diagnosis and prognosis**
Difficulty recalling diagnosis^26^ No information available about diagnosis (following CMA or ES)^15^, ^30^ Prognosis around learning disability^15^, ^26^, ^27^ Prognosis around spectrum disorder^15^, ^18^, ^26^ Prognosis around what child will look like/be like^15^, ^27^, ^28^, ^33^ Whether baby will survive^30^ Uncertainty around whether condition was inherited^15^, ^18^, ^26^, ^27^ Variants of uncertain significance found (VUS)^18^, ^26^, ^27^, ^28^, ^29^, ^30^, ^34^	‘I can't remember which letter or number it was— it was 22 or something’. *[Patient quote]* ‐^26^ In two cases women said that they had not received enough information. One of these cases involved an uncertain chromosome result where no accurate information was available. *[Findings]* ‐^30^ ‘Since I had this uncertain microarray result … if anything happens to him in the future … that will always pop up in my mind…. You just have to have a “wait and see” attitude…. I'm a lot more vigilant’. (Participant 8) ‐^[^ ^14^ ^]^ One woman who terminated a pregnancy diagnosed with a de novo DiGeorge deletion said: ‘We still grapple with this because it is very much a spectrum of severity, very, very hard to predict what the outcome would be…. So that was very, very difficult for us because it made assessing our choices really hard’. *[Patient quote]* ‐^15^ ‘I was upset, because they could not tell me exactly how high the risk of developing the clinical features was. I just sat there stared at the geneticist and asked what it was, and if it was dangerous’. *[Patient quote]* ‐^33^ Many women and their partners expressed uncertainty and lack of control over the situation. Two women expressed distress at not knowing if their unborn child would live or die. *[Findings]* ‐^30^ Genetic testing of biological parents confirmed whether the variant was inherited or de novo. If the variant was inherited from a parent who had no clinical presentation, participants reported being reassured by their providers that the baby likely develops typically as well. *[Findings]* ‐^18^ Two people described getting the result of unknown significance (VUS). The second couple found the uncertainty difficult to deal with: ‘You never think a doctor's going to go, phew, don't know what it is’. *[Patient quote]* ‐^30^
**Accuracy of online information**^15^, ^27^	‘We did a little bit of research online, but when you look online, you ‐ it's just nonsense. I mean some are true, some are false’. ‐ [patient quote]^27^
**Health professionals’ uncertainty**^15^, ^27^, ^30^	‘You know, they're telling me there's something wrong, but they can't tell me what…. We wanted to know what that would mean for our son in the future. And they really couldn't tell us’. ‐ [patient quote]^15^ ‘I assume nobody really knows and because they don't know they can't tell me’. ‐ [patient quote]^30^

##### Testing procedure

Seven studies described uncertainty stemming from the testing procedure and the possible results. 6, 15, 18, 19, 26, 30, 31 Some women were uncertain about what a microarray test was.^15^, ^30^ In one study, a participant described not being aware of the possibility of receiving inconclusive results,^18^ and there was uncertainty about how the test results would be delivered and by whom.^6^, ^19^

##### Results: Including the diagnosis and prognosis

In two studies,^15^, ^30^ participants described not receiving enough information following their results due to the unavailability of accurate information, or HCPs limiting the amount of information they fed back due to concerns around upsetting the participants. In one study, some participants had difficulty recalling the result they were given.^18^ In seven studies, participants received a VUS following CMA, which led to uncertainty.^18^, ^26^, ^27^, ^28^, ^29^, ^30^, ^34^ VUS often prompted additional stress as participants thought genetic testing would give them more answers, instead of creating more uncertainty. Participants struggled with the lack of information surrounding what their child would look and be like, as well as the severity of the condition.^18^ In one study, many women and their partners expressed distress at not knowing if their unborn child would live or die.^30^

Four studies described uncertainty around whether the variant was inherited or de novo following receipt of an uncertain result, and participants felt a sense of reassurance and relief on discovering a hereditary variant in a parent with no clinical presentation.^15^, ^18^, ^26^, ^27^ Rubel et al described one participant who acknowledged that inheritance does not completely remove the risk of phenotypic expression, even with a ‘normal’ parent, and described parental testing as providing a ‘false sense of security’.^27^

##### Online health information

Two studies^15^, ^27^ highlighted that parents often searched for further information online, but were not clear as to whether the information they found was accurate, hence it did not resolve their uncertainties.

##### Healthcare professionals' own uncertainty

Three studies indicated that uncertainty for parents could arise from HCPs lack of knowledge or uncertainty around the diagnosis or condition identified.^15^, ^27^, ^30^ Participants also described receiving conflicting information from different HCPs.^15^ In some cases, participants were unable to obtain any further information about their result from HCPs, as they assumed their HCP did not know anything further. One participant expressed shock that their clinician was unable to provide any certainty about the meaning of their result.^30^

#### 3.1.2 Impact of uncertainty

Findings relating to the impact of the uncertainty were either about the (a) clinical impact or (b) emotional impact.

##### Clinical impact

Uncertainty could affect clinical decision‐making and future practical plans. Five studies showed that participants found making clinical decisions based on uncertain test results challenging, in particular whether to continue or terminate the pregnancy.^6^, ^15^, ^18^, ^27^, ^30^ This included studies where patients had received a negative ES results,^6^ a finding of a deletion or duplication syndrome,^15^, ^18^ and a VUS.^27^, ^30^ Having uncertainty surrounding the prognosis for the baby, as well as the general lack of information about the future, made it difficult for participants to make decisions. For example, Bernhardt et al found that many women felt they needed more support when working out the next steps for their pregnancy.^15^ In addition, participants struggled to deal with having to make decisions in such a short amount of time. Werner‐Lin et al found that participants felt burdened with the pressure of managing this complex information, while dealing with their anxiety, within the limited time period.^18^

Participants felt that they were unable to plan for the future with a lack of information or resources to alleviate their concerns or answer their questions.^27^, ^28^ Furthermore, there were practical implications of uncertainty, particularly around preparing for the upcoming birth when the prognosis was uncertain, with one parent explaining that it took ‘two or three more months after the tests to even buy the crib’.^15^

##### Emotional impact

The emotional impact of uncertainty could create feelings of worry, affect relationships and could continue to affect parents after the child was born. Participants from six studies reported feeling shocked and worried on receiving uncertain results.^15^, ^18^, ^19^, ^27^, ^30^, ^33^ Participants across three studies^15^, ^18^, ^27^ described wishing that they did not have the information about uncertain results, which Bernhardt et al and Rubel et al referred to as ‘toxic knowledge’.^15^ ^27^ This emotional overload was often replaced with ongoing anxiety, which was reported in nine studies,^6^, ^15^, ^18^, ^19^, ^26^, ^27^, ^28^, ^33^, ^34^ along with lingering worries and uncertainties. In addition, Halliday et al found state anxiety scores to be slightly higher in women who had received an extended analysis report, which included VUS, compared to a targeted analysis, although this difference was not statistically significant.^34^

Halliday et al found that decisional regret scores regarding the decision to undergo genetic testing, were higher for participants who chose to receive VUS compared to those who did not choose to receive VUS results.^34^ This was also reflected by Desai et al, who reported that participants who received VUS felt less satisfied with their decision 36 months after birth, compared to those who received normal and clearly abnormal results.^29^

Five studies reported participants feeling overwhelmed by the future as well as a lack of control over the uncertain situation following uncertain results.^18^, ^27^, ^30^, ^32^, ^33^ Many participants questioned what would happen next as they struggled to comprehend the information and look to the future. Furthermore, Hillman et al found that participants were concerned that the issue of uncertainty could be repeated in a future pregnancy.^30^ However, in one case, a couple expressed that uncertain results still provided extra information that could be beneficial for their future, stating that ‘at least we know more, we are going to be prepared’.^30^

One study described the impact of uncertain results on the relationship between the pregnant women and their partners.^6^, ^15^, ^18^ There could be conflicting opinions between partners, with one wanting to discuss the pregnancy with friends and family, and the other preferring to keep the pregnancy private. However, couples also found that these difficult experiences could strengthen their relationship as the long, emotional conversations resulted in an ‘aligning of their priorities’.^18^

The emotional impact of uncertainty could continue after the child was born. One study described how mothers would be in a state of ‘watchful waiting’ as they would monitor the health and development of their child, scrutinise their child's appearance, and make comparisons against their unaffected children.^28^ One participant commented ‘when things weren't as advanced as my first daughter, we would question, “do you think it's that?”’.^28^

#### 3.1.3 Managing uncertainty

Parents had differing levels of tolerance when it came to receiving uncertain information. Some parents reported wanting to know as much information as possible despite the potential for receiving uncertain results, whilst other parents did not want to receive such information.^31^, ^32^, ^33^, ^34^ Three studies reported the experience of participants who were not additionally concerned by receiving an uncertain result.^17^, ^26^, ^28^ For example, one participant, described by Werner‐Lin et al, explained how she did not give the VUS a second thought after birth.^28^ The majority of participants, however, reported not receiving as much information as they wanted, which they felt prevented them from gaining definitive answers and ‘grasping the significance’ of their results.^26^

In dealing with uncertainty, parents were found to seek support and further information, whether this was through speaking with a HCP such as a genetic counsellor, their friends and family, other parents or searching for information online.^6^, ^15^, ^17^, ^18^, ^19^, ^26^, ^27^, ^28^, ^30^ The majority of participants appreciated support from their HCP, including a referral to a genetic counsellor for emotional support, particularly as uncertain results could need longer, more specialised or more frequent counselling.^6^, ^15^ Participants also relied on friends and family, especially during the period of time straight after receiving results when they were most distressed and scared. However, Werner‐Lin et al reported that participants sometimes did not want to share the uncertain information with family members, for fear of stigma towards their child after birth and a lack of understanding from others with one patient commenting ‘My dad would treat [child] differently even though the results don't say anything definitive’.^28^ Participants also reported the utility of speaking to other parents in similar situations and this was mainly achieved on the internet, through online communities and advocacy groups. Furthermore, Wou et al found that many participants would have liked to be connected with another family with a similar experience, for mutual support and understanding.^6^

One study illustrated how uncertainty could also be managed as a couple, with both the pregnant woman and her partner playing an important role in the process. For example, Werner‐Lin et al found that, within a couple, the pregnant woman often acted more as a seeker of information, while her partner provided emotional support, to help with decision making.^18^

Three studies reported how participants' spoke of remaining hopeful.^15^, ^28^, ^30^ They remained hopeful that they would eventually find enough information to make informed decisions and hoped that their test results could be used by researchers to provide answers for women in the future.^15^, ^30^ In addition, Werner‐Lin et al found couples remained hopeful and stayed positive after the birth at the same time as closely watching the progress and development of their child.^28^

## 4 DISCUSSION

With the growing availability of new prenatal genomic tests such as ES in clinical practice,^36^ prospective parents are more likely to face uncertain test results. This review provides a synthesis of 14 studies on pregnant women and their partners' experiences of uncertainty in the prenatal setting. Our findings highlight how uncertain prenatal results can affect parents in different ways. Some parents were surprised to receive uncertain findings and struggled to make clinical decisions based on an uncertain prognosis in a limited timeframe. For others, even uncertain information is better than no information. Our findings complement a recent narrative review which found that patients respond to uncertainty in different ways, based largely on their own general sense of optimism and tolerance for personal ambiguity as well as their past experiences with uncertainty, reproduction and family planning.^12^

Detecting uncertain CMA/ES results raises significant ethical considerations, in particular how to balance the potential harm to a woman or her foetus with the rights associated with patient autonomy and whether it is ethically justifiable to withhold any test result information from a patient.^37^ How these competing rights are viewed is likely to differ across countries. For example, in the United Kingdom the policy is that incidental findings and VUS and low penetrance neuro‐susceptibility loci are generally not reported.^38^ In the United States, the type and amount of information reported varies depending on the policy of the laboratory performing the analysis. Recent ACMG guidelines on the use of prenatal ES advocate that laboratories should have clear policies for what types of variants, including VUS, will be reported and recommends that pre‐test counselling includes discussion of the potential to identify VUS as well as adult‐onset diseases in the fetus.^39^

Health professionals have an important role in uncertainty management in a prenatal setting. Previous studies suggest that clinicians can feel uncomfortable providing uncertain CMA results.^40^, ^41^ This can be particularly challenging for clinicians without specialist training in genetics.^42^ The lack of educational resources to support patients is also an issue.^43^ Uncertain findings can have a negative impact on the doctor‐patient relationship, as parents sometimes react angrily when they are struggling to make decisions about the pregnancy.^35^ Whilst patients might be informed during pre‐test counselling of the possibility of receiving uncertain findings, it may be that the reality of such findings is not being properly considered prior to testing.^15^ It has been suggested that clinicians could perhaps discuss with parents their tolerance for ambiguity as part of pre‐test genetic counselling, to ascertain whether information that is uncertain will be useful or problematic for them personally.^15^, ^44^ Biesecker et al suggests that examining patients' tolerance of uncertainty, resilience and optimism alongside their expectations about genomic testing, may help to identify those more likely to appraise uncertainty as a threat, and to alleviate negative responses.^45^ In some settings, parents have been offered the choice between ‘targeted’ and ‘extended testing’ whereby CNVs with incomplete penetrance and VUS are reported.^32^, ^34^ Furthermore, research in this area would be valuable.

Finally, another area for future research relating to uncertainty is parent experiences and views of the reanalysis when there is a VUS or no findings. Previous research with clinicians, scientists, genetic counsellors and patient groups/charities, has found that patient representatives supported reinterpretation of results over time, more so than other participant groups.^35^

### 4.1 Strengths and limitations

Strengths include the systematic and rigorous approach taken to identify and appraise the studies, that all were high quality studies, and the inclusion and integration of results from qualitative and quantitative research which provides rich data on parents' experiences.

Limitations include that the sample is predominantly comprised of white, educated participants. Therefore, the findings lack the perspectives of minority ethnic groups and those from lower educational backgrounds, both who experience inequity in access to healthcare‐systems and disparities in understanding prenatal testing options.^46^, ^47^ The experiences of partners are underrepresented in this study, accounting for only 26% of the total sample. Finally, only two studies investigated the experiences of those undergoing ES, which makes it difficult to make comparisons between the experiences of parents' undergoing ES and CMA.

## 5 CONCLUSION

**FIGURE 3:**
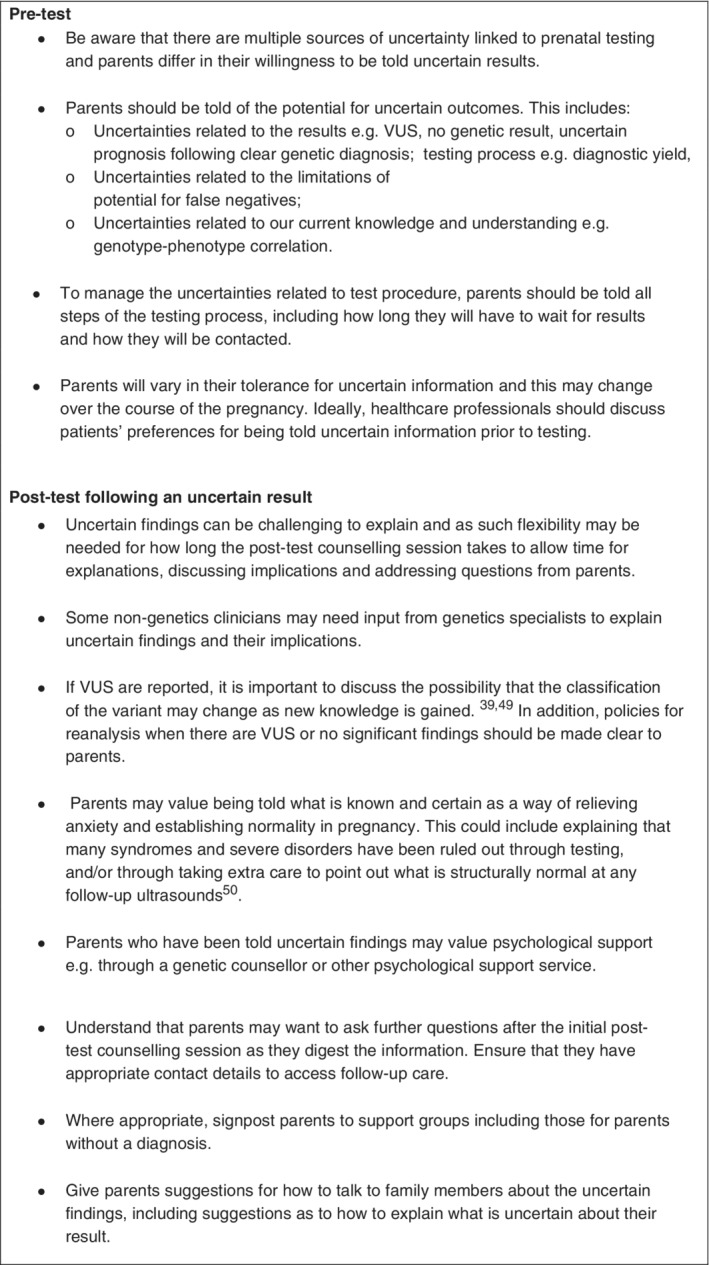
Recommendations for pre‐ and post‐test counselling about uncertainty

The findings of this review highlight the different types of uncertainties that prospective parents experience in the prenatal testing setting, and the implications of these uncertainties. Whilst many of the uncertainties relate to our current knowledge and understanding of genotype‐phenotype correlation, there are some uncertainties that can be managed during pre or post‐test counselling for example, parents not aware that the test could reveal uncertain results. Moreover, we identified evidence of good‐practice when managing uncertain results for example, additional support. In light of these findings, we have developed a set of recommendations for HCPs as a guide for best practice when offering prenatal testing (Figure 3).^39^, ^48^, ^49^ Whilst there are some guidelines on mitigating for these issues, further research should look to explore optimal ways of managing uncertainty in the prenatal setting to minimise the potential for patient harm.^50^

## CONFLICT OF INTEREST

The authors declare that they have no conflicts of interest.

## AUTHOR CONTRIBUTIONS

C.L. conceived the study. E.H. and M.H. identified and appraised the potential studies. E.H. synthesised the studies. E.H., M.H. and C.L. developed a codebook and coded the studies. E.H., J.H., M.H. and C.L. analysed the data. E.H. and J.H. drafted the paper. L.C., M.H. and C.L. revised the draft paper.

## Data Availability

Research Data are not shared.
